# Non-Invasive Electroretinogram Recording with Simultaneous Optogenetics to Dissect Retinal Ganglion Cells Electrophysiological Dynamics

**DOI:** 10.3390/bios13010042

**Published:** 2022-12-28

**Authors:** Eunji Hong, Christopher Glynn, Qianbin Wang, Siyuan Rao

**Affiliations:** 1 Department of Biomedical Engineering, University of Massachusetts, Amherst, MA 01003, USA; 2 Institute for Applied Life Sciences, University of Massachusetts, Amherst, MA 01003, USA; 3 Neuroscience and Behavior Graduate Program, University of Massachusetts, Amherst, MA 01003, USA

**Keywords:** non-invasive recording, biosensors, neural electrophysiology, neurodegeneration, optogenetics, electroretinogram

## Abstract

Electroretinography (ERG) is a non-invasive electrophysiological recording technique that detects the electrical signaling of neuronal cells in the visual system. In conventional ERG recordings, the signals are considered a collective electrical response from various neuronal cell populations, including rods, cones, bipolar cells, and retinal ganglion cells (RGCs). However, due to the limited ability to control electrophysiological responses from different types of cells, the detailed information underlying ERG signals has not been analyzed and interpreted. Linking the features of ERG signals to the specific neuronal response will advance the understanding of neuronal electrophysiological dynamics and provide more evidence to elucidate pathological mechanisms, such as RGC loss during the progression of glaucoma. Herein, we developed an advanced ERG recording system integrated with a programmable, non-invasive optogenetic stimulation method in mice. In this system, we applied an automatic and unbiased ERG data analysis approach to differentiate a, b wave, negative response, and oscillatory potentials. To differentiate the electrophysiological response of RGCs in ERG recordings, we sensitized mouse RGCs with red-light opsin, ChRmine, through adeno-associated virus (AAV) intravitreal injection. Features of RGC dynamics under red-light stimulation were identified in the ERG readout. This non-invasive ERG recording system, associated with the programmable optogenetics stimulation method, provides a new methodology to dissect neural dynamics under variable physiological and pathological conditions in vivo. With the merits of non-invasiveness, improved sensitivity, and specificity, we envision this system can be further applied for early-stage detection of RGC degeneration and functional progression in neural degenerative diseases, such as glaucoma.

## 1. Introduction

Neural degeneration diseases in the vision system, such as glaucoma, lead to irreversible blindness via progressive retinal ganglion cell (RGC) loss and optic nerve damage [1,2]. Effective and timely intervention is hampered by inadequate diagnostic methods [3]. In most cases, the early onset of symptoms remains undetected until the advanced stages and presentation of serious structural damage and vision loss [4]. Early detection of abnormal neural function is important for intervention within the critical period of neural degeneration [5]. Existing bioimaging techniques, such as fundus photography and optical coherence tomography (OCT), can assess pathological progression with morphological information of retinal tissues [6,7]; however, such imaging-based techniques cannot detect detailed information to reflect neural function change during degeneration, which disrupts cellular electrophysiological activity [8]. There is an urgent need to assess neural electrophysiological activities in the vision system and reveal the mechanism of neural degeneration.

As a non-invasive electrical recording technique, electroretinography (ERG) has been used in ophthalmologic diagnosis to identify glaucoma [9,10]. In prior glaucoma research, conventional ERG systems for rodent experimental subjects consisted of a working electrode placed on the surface of the cornea, a reference electrode under the skin on the forehead, and a ground electrode fixed in the tail [11]. This type of three-electrode system is designed to detect the electrical potentials generated from the vision system upon optical stimulus. Generally, ERG signals are composed of three prominent features at low frequency and one feature at high frequency originating from the outer retina. At low frequencies, an initial negative deflection (a-wave) and a subsequent positive peak (b-wave) followed by a photopic negative response (PhNR) reflect contributions from photoreceptors, light-induced bipolar and Muller cells, and retinal ganglion cells [11], respectively. At high frequencies, there are oscillatory potentials (OPs) [11]. ERG relays a composite signal from each of these contributing neural populations. Analysis and interpretation of ERG signals are challenged by differentiating each of these signal contributions [12,13]. Additionally, in vivo electrical recording noises contaminate the featured waves at low frequencies. The existing ERG systems and the signal analysis approaches are insensitive to subtle changes in neural electrophysiological activities related to early pathological development.

The intrinsic structure of the vision system allows light transmission into deep tissues, offering a noninvasive method for optical stimulation and investigation. Differentiated contributions to ERG signals from selectively triggered cell populations can provide detailed insight into neural electrophysical dynamics. As an important toolset in neuroscience study, optogenetics offers a method to modulate neural activity with optical stimuli [14,15]. Specific types of neurons can be sensitized by viral vector delivery of photosensitive ion channel (opsin) genes and, subsequently, express opsins triggered by select wavelengths of light. Recently, there has been promising progress in the application of optogenetic therapy to partially reverse neurodegenerative damage and restore vision in a patient suffering from retinitis pigmentosa [16,17]. More advanced red-shifted opsins have been developed for non-invasive optical modulation of neural activity using longer wavelengths of light towards deep brain regions in rodent and non-human primates [18,19,20]. Mice, known as dichromats, offer the unique advantage under red-light optical stimulation in the vision system to differentiate electrophysiological responses of neural populations that lack endogenous sensitivity to long wavelengths [21].

In our study, we specifically choose ChRmine, a type of red-shifted-channelrhodopsin with high photocurrents and light sensitivity to understand RGC electrophysiological dynamics [18,22]. To provide insight into detailed cellular electrophysiological dynamics, we custom-developed a non-invasive ERG recording system associated with a programmable optogenetics stimulation component in mice. We also established a set of automatic and unbiased data analysis algorithms to distinguish detailed ERG features, including a-wave, b-wave, and negative response (NR) “low-frequency” components and OPs “high-frequency” components. We sensitized RGCs with adeno-associated virus (AAV) delivery of red-shifted opsin, ChRmine, and systematically analyzed the ERG responses of RGCs to a series of differently colored optical situations. We discovered that high-frequency OPs are directly associated with RGC electrophysiological activity. This provides direct evidence to demonstrate RGC’s contribution to ERG signals and a new diagnostic target for RGC-related diseases, such as glaucoma.

## 2. Materials and Methods

### 2.1. Animals

All experimental protocols were approved by the Institutional Animal Care and Use Committee (IACUC) at the University of Massachusetts Amherst and were consistent with local, state, and federal regulations as applicable. For the care and use of laboratory animals, C57BL/6J mice at 6–8 weeks old (20–25 g), originally sourced from Jackson Laboratory (JAX), were used for ERG recordings and AAV injections. All mice were maintained (maximum of 5 mice per cage) and were on a reverse 12 h light/dark cycle with food and water ad libitum.

### 2.2. AAV Packaging

Recombinant AAV vectors were produced following the plasmid co-transfection method as previously described [23]. Briefly, HEK-293T cells were transfected by retro helper plasmid (Addgene plasmid #81070), AAV rep-cap plasmid to produce viral capsid AAV2/2 (Addgene plasmid #104963), and cis-transgene plasmid to produce ChRmine-mScarlet or mScarlet under the control of neuron-specific CaMKIIα promoter with polyethyleneimine (PEI) (Linear, MW 25,000, Polysciences, Warrington, PA, USA). pAAV-CaMKIIa-ChRmine-mScarlet and pAAV-CaMKIIa- mScarlet plasmids were gifts from Karl Deisseroth (Addgene plasmid #130991 and #131000). Iodixanol gradient ultracentrifugation with Beckman Coulter Optima XL-70 and VTi 50.1 rotor was used to purify virus particles. Viral titers were measured by qPCR according to the manufacturer’s instructions (Addgene, Watertown, MA, USA). Viral stocks were kept at −80 °C.

### 2.3. Intravitreal AAV Injection

Mice were anesthetized via intraperitoneal injection of a ketamine hydrochloride (100 mg kg^−1^, NADA# 043-304, Zoetis, Spain) and xylazine (10 mg kg^−1^, NADA# 139-236, Akorn, Lake Forest, IL, USA) mixture and placed on a heating blanket to maintain their body temperature. One drop of 1% tropicamide ophthalmic solution, USP (NDC 17478-102-12, Akorn, USA) was applied to the cornea before injection. A small hole was punctured by a 30G needle near the peripheral retina behind the ora serrata. A total of 1 µL of AAV2/2-*CaMKIIα: ChRmine-mScarlet* (3.03 × 10^12^ GC/mL), AAV2/2-*CaMKIIα: mScarlet* (2.46 × 10^12^ GC/mL) or phosphate buffer saline (PBS; Cat# P3813, Sigma-Aldrich, St. Louis, MO, USA) were injected into the mouse vitreous body by using a pulled-glass micropipette. Antibiotic ointment was applied to the cornea surface post-injection.

### 2.4. ERG Recording

Mice from each group were adapted to the dark environment for at least 10 min before the ERG recording and anesthetized with a 100 mg/kg ketamine hydrochloride and 10 mg/kg xylazine mixture by intraperitoneal injection. One drop of 1% tropicamide ophthalmic solution was applied to the cornea surface to dilate pupils. Dark adaptation or a red-light environment was maintained throughout the recording. A four-lead 5 mm RGB LED and a red-light LED of 625 nm wavelength (Cat# M625F2, Thorlabs, Newton, NJ, USA) were used for color-induced and optogenetic stimulation ERG recording, respectively. Two 0.5 mm coiled shape silver wires (Cat# 265586, Sigma-Aldrich, USA) were placed on the corneal surface of the test eye (serving as the “working electrode”) and the reference eye (serving as the “reference electrode”). Signals from the working electrode and the reference electrode were automatically subtracted (“a–b” differential mode) with an extracellular amplifier (Model DAM50, World Precision Instruments, Sarasota, FL, USA). Recorded ERG signals were amplified (10,000×), filtered (1–1000 Hz), and digitized at 10,000 Hz (Model DI-1100, DATAQ Instruments, Akron, OH, USA). Flash ERGs were recorded for 5 flashes with a 50 ms flash duration and an interval of 4.95 s delivered to the test eye.

### 2.5. ERG Featured Signals Dissection

Home-developed Matlab (2021a)-based programs were used to sort ERG features using fast Fourier transform (FFT) and inverse fast Fourier transform (IFFT). A frequency below 50 Hz was defined as the low-frequency components of ERG, while a frequency higher than 65 Hz was defined as the high-frequency components. The low-frequency components were used to quantify the amplitude of the a-wave (difference between the a-wave peak and baseline), b-wave (difference between the b-wave peak and baseline), and NR (difference between the NR peak and baseline). The high-frequency components were used to quantify the amplitude of OPs (the maximum difference between the positive and negative peaks).

The algorithm employed in this study identifies the signal from spike prominence and performs a subsequent analysis of each extracted waveform. The procedures can be described in the following steps: (1) generate a threshold from resting potential mean and variance; (2) index and extract spikes from data; (3) segregate low and high frequency components; (4) register amplitudes of a-wave, b-wave, NR (low frequency), and OPs (high frequency) components. The continuous wavelet transform (CWT) was performed using Matlab Wavelet Toolbox. All the programs were run on a desktop computer Dell Precision 7920, Intel Xeon, 192 GB RAM.

### 2.6. Immunostaining

For immunostaining, all of the mice were euthanized using an intraperitoneal injection of Fatal-Plus (78 mg kg^−1^, Vortech Pharmaceuticals, Dearborn, MI, USA) and transcardiacally perfused with ice-cold PBS followed by 4 wt% paraformaldehyde (PFA; Cat# P6148, Sigmai-Aldrich, USA) solutions in PBS. After perfusion, eye tissues with optic nerve heads were dissected out and postfixed in 4 wt% PFA PBS solutions overnight at 4 °C. For retinal cryosections, tissues were cryoprotected in 30 wt% sucrose (Cat# S9378, Sigma-Aldrich, USA) in PBS for 24 h before freezing in Tissue-Tek Optimal Cutting Temperature (OCT) compound (Cat# 4583, Sakura Finetek, Torrance, CA, USA). Then, 20 μm-thick cryosections were mounted on SuperFrost slides (Cat# 12-550-15, Fisher Scientific, Hampton, NH, USA). For whole-mount retinas, retinal tissues were dissected out and rinsed in PBS.

Antibodies were diluted in a blocking solution containing 0.5 *v*/*v*% Triton-X (Cat# X100, Sigma-Aldrich, USA) and 1 wt% bovine serum albumin (BSA; Cat# A9647, Sigma-Aldrich, USA) in PBS. Retinal cryosections were incubated overnight at 4 °C in the primary antibody (Guinea pig anti-RBPMS, 1:400, Cat# A008712, Raygene) and 2 h in the secondary antibody (Alexa Fluor^®^ 647 AffiniPure Donkey Anti-Guinea Pig IgG (H + L), 1:200, Jackson ImmunoResearch Labs, USA) at room temperature, while whole-mount retinas were incubated for 3 days in primary and 5 h in secondary antibody at 4 °C. Retinal cryosections were mounted with 4′,6-diamidino-2-phenylindole (DAPI)-containing Fluoromount-G (Cat# 010020, Southern Biotech, Birmingham, AL, USA), and whole-mount retinas were mounted with Fluoromount-G (without DAPI; Cat#010001, Southern Biotech, USA). Confocal images were collected with a Leica SP2 laser scanning microscope. The processing of image stacks was done using Fiji software.

### 2.7. Data Analysis

Data were analyzed by GraphPad Prism software. The data were plotted as mean ± standard deviation (s.d.). Statistical significances were carried out using paired or unpaired one-way analysis of variance (ANOVA) and Tukey’s multiple comparisons test. Significances are indicated as follows; * *p* < 0.05, ** *p* < 0.01, *** *p* < 0.001, and **** *p* < 0.0001.

## 3. Results and Discussion

### 3.1. A Custom-Designed ERG Recording System Associated with Optical Stimulation and Algorithmic Signal Feature Classification

**Figure 1:**
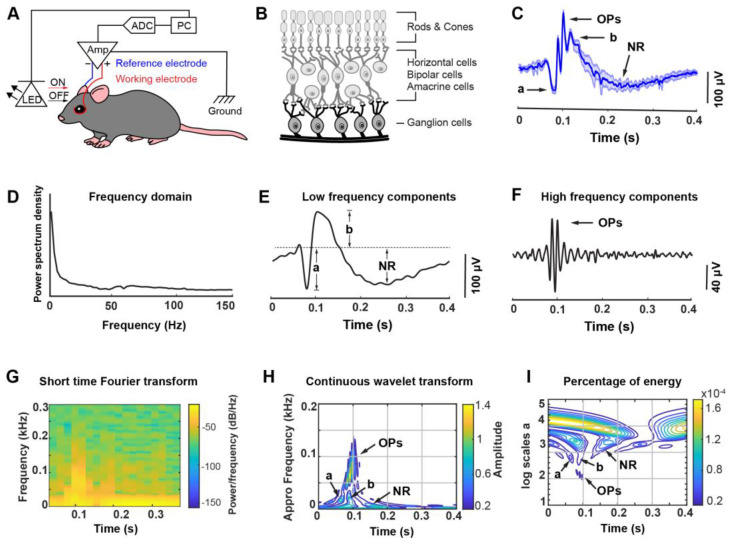
A custom-designed ERG recording system associated with a programmable optogenetics stimulation device. (**A**) Schematic illustration of the device configuration for the stimulation and recording in mice. (**B**) Schematic illustration of the layered structures of cells in the mouse retina. (**C**) A representative scotopic flash ERG waveform recorded with the custom-designed device from the same mouse eye (solid line: mean, shaded area: standard deviation (s.d.), n = 5 repeated measurements. The duration of optical stimulation was 50 ms, with 4950 ms latency period). The amplitude plot in the frequency domain after Fourier transform (**D**), low-frequency components (less than 50 Hz) (**E**), and high-frequency components (higher than 65 Hz) (**F**) from the scotopic flash ERG response in (**C**). The short-time Fourier transform (**G**), the continuous wavelet transform (**H**), and the percentage of energy analysis (**I**) of the scotopic flash ERG response in (**C**). LED, light-emitting diode; Amp, amplifier; ADC, analog-to-digital converter; PC, personal computer; Ground, ground electrode; a, a-wave; b, b-wave; OPs, oscillatory potentials; NR, negative response.

We first modified the three-electrode ERG system by relocating the reference electrode on the contralateral eye of the same experimental subject to account for noise contributions from eye tissues. Two coiled-shape silver wires were placed on the corneal surface of the eye: a working electrode (electrode “a”) on the test eye and a reference electrode (electrode “b”) on the contralateral eye (Figure 1A). The ERG signals were collected with 10,000× amplification. Signals were filtered between 1 and 1000 Hz with an extracellular amplifier and digitized at 10,000 Hz with a data acquisition system (see Methods). The ERG signals were recorded using the differential mode (“a–b”) of the amplifier to eliminate background noise and crosstalk from the reference eye. A programmable LED light source was placed in front of the test eye at a fixed distance.

To validate the functionality of our system, we conducted ERG recordings in wild-type mice with white-light scotopic flash stimulation (intensity of 1.9 mW) (Figure 1B,C). Representative ERG signals with magnitudes of 266 ± 3 µV and a duration of 50 ms were detected after scotopic flash stimulation. The scotopic flash ERG response consists of the initial a-wave, a subsequent b-wave with the oscillatory potentials (OPs) on the rising part of the b-wave, and a terminal slow negative response (NR). The a-wave is the first cornea-negative deflection, which mainly reflects the photocurrents generated by the rod and cone receptors. The b-wave is the largest component of the scotopic flash ERG, with a cornea-positive deflection following the a-wave. The b-wave is primarily generated by the depolarization (ON) of bipolar cells. The scotopic ERG responses display large OPs, which reflects the neuronal interactions in the inner plexiform layer between bipolar cells, amacrine, and ganglion cells. In the conventional ERG recording, the reference electrode was placed under the skin on the forehead to cancel out background noise [24]. In our modified ERG recording system, we used two corneal electrodes to make this recording system completely non-invasive and provide a more sensitive representation of the complex wave signal. The differential amplifier circuit between the working and reference electrodes can further minimize variation between tissue locations and increase the measurement sensitivity.

Then we developed a set of algorithms to automatically and unbiasedly classify ERG signal features. We applied fast Fourier transformation and inverse fast Fourier transform to derive frequency-dependent signals and quantify the amplitudes of the corresponding waves, respectively (Figure 1D–F). Fourier transform of the ERG signal revealed two distinct amplitude ranges in the frequency domain (Figure 1D): a low-frequency signal (below 50 Hz) with slow components, a-, b- and NR waves, and a high-frequency signal with a peak around 65–75 Hz with fast components, e.g., OPs (Figure 1E,F). At low frequencies, a-, b-wave, and NR were detected with amplitudes of 93 ± 11 µV, 59 ± 23 µV, and 88 ± 15 µV, respectively (Figure 1E). At high-frequency regions (larger than 65 Hz), OPs were detected with an amplitude of 159 ± 8 µV in the primary ascending phase of the b-wave (Figure 1F). To further elucidate the frequency-dependent features of each wave with a better temporal resolution, the short time Fourier transform (STFT) and continuous wavelet transform (CWT) were applied to extract the additional information of represent time and frequency marginals (Figure 1G–I). We observed a high-frequency component located between 80 ms and 120 ms with a power value of −22 dB/Hz to −25 dB/Hz (Figure 1G). The A-wave has a low frequency and a low energy band of 12 to 26 Hz, with amplitude concentrated at 18 Hz, and the b-wave has a slightly higher frequency and energy band that exists in the range of 13–33 Hz, with amplitude concentrated at 22 Hz. The OPs formed three main distinct clusters centered at 66 Hz, 73 Hz, and 85 Hz with small amplitudes and short durations, which reflect weak electrical signals. With a slow response, NR showed a very large energy band in the energy scalogram (Figure 1H,I). The combination of this non-invasive ERG recording system and automated signal analysis algorithm provide a platform to efficiently dissect major components of ERG signals and lay a foundation for the future investigation of cell-specific contributions to ERG features.

### 3.2. ERG Responses under Different Color Light Stimulation

**Figure 2:**
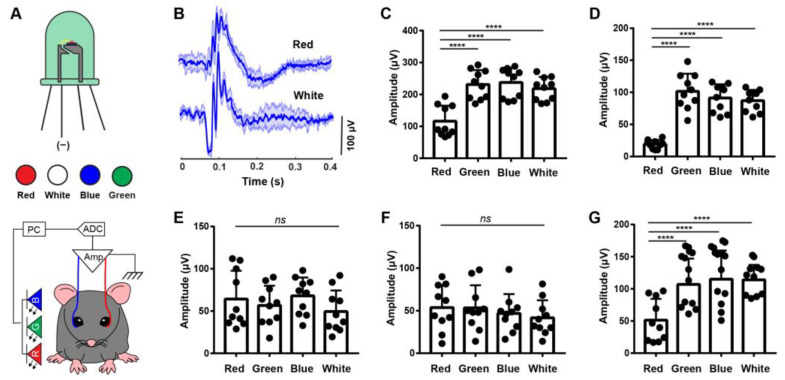
Scotopic flash ERG responses under different color light stimulation. (**A**) Schematic illustration of ERG recording system with an RGB LED light source. (**B**) Representative scotopic flash ERG waveforms under red- and white-light stimulation (solid line: mean, shaded area: s.d., n = 4 independent eye measurements from 2 mice). (**C**) Quantitative comparison of the ERG overall maximum response for different color light stimulations. The amplitude comparison of a-wave (**D**), b-wave (**E**), NR (**F**), and OPs (**G**) from the scotopic flash ERG responses under red-, green-, blue-, and white-light stimulations. N = 10 eyes. PC, personal computer; ADC, analog-to-digital converter; Amp, amplifier. ns: not significant. **** *p* < 0.0001.

Mice, as dichromats, have different sensitivities to different colors. Accordingly, we hypothesize that different color light stimulation can evoke specific electrophysiological responses. To test this hypothesis, we designed a programmable LED light source that can produce three primary additive colors: red (635 nm), green (520 nm), and blue (460 nm) (RGB light source) and assessed ERG signal features variation to determine the retinal response to each color (Figure 2A). The patterned red, green, blue, and white light stimulation can be programmed and controlled with specific parameters, i.e., a duty cycle with 50 ms duration and a period of 5 s. With the benefit of the remote control of the light stimulation, the light source and subject position can be fixed. This minimizes the environmental variation on the same experimental subject and allows a more accurate comparison of ERG signals among different color light stimulations.

We found substantial differences between the scotopic flash ERG responses under red and white light stimulations (Figure 2B). Compared to the white light response, the ERG signals of red-light stimulation showed a lower amplitude a-wave and a smaller high-frequency component (OP) on the b wave, while b-wave and NR amplitudes were comparable. To comprehensively dissect the response to each color of light, we then quantified the overall maximum response, a-wave, b-wave, OPs, and NR upon red-, green-, blue- and white-colored light stimulation (Figure 2C–G). In all examined mouse eyes, the photoreceptor-mediated scotopic a-wave exhibited a significantly lower amplitude (19 ± 7 µV) than other colored-light-triggered a-waves. The a-wave amplitudes upon green-, blue-, and white-light stimulation were 101 ± 27 µV, 91 ± 21 µV, and 87 ± 17 µV, respectively (Figure 2D). Red-light-evoked b-wave and NR amplitudes showed no significant difference compared to other colored light stimulation (Figure 2E,F). The amplitude of red-light-evoked OPs was dramatically reduced compared with those of other colored light stimulations, shown as 51 ± 33 µV for red light stimulation, 106 ± 40 µV, 115 ± 44 µV, and 114 ± 23 µV for green, blue, and white light stimulations, respectively (Figure 2G). The dramatically reduced a-wave and OPs components under red light stimulations produced overall ERG signals with significantly lower amplitudes (Figure 2C).

Mice are considered insensitive to red light because of the lack of long-wavelength cones in their retinas. In mouse husbandry and experimental conditions, red light is widely used to keep mice in a “dark” environment while allowing humans to operate. However, based on sorted ERG signals upon different colored light stimulation, we found that the red-light-evoked b-wave and NR have similar amplitudes to the signals triggered by other colored light. The prior sorted ERG signals under different color light stimulation exhibited similar b-wave and NR contributions under red light as other colors, suggesting the cells contributing to b-wave and NR signals are activated by red light as well. This phenomenon can be explained with the principle of univariance, which indicates that a large intensity of long-wavelength photons can activate cones as well. This suggests that light intensity is another important factor in the study of color-dependent cell activation.

This electrophysiological evidence might indicate that mice are not completely red-light blind, as there are still low-frequency ERG components. We used to consider the leading edge of the scotopic a-wave to represent rod photoreceptor cells, which do not mediate color vision [25,26,27]. Surprisingly, the scotopic rod-driven a-wave collected in our system shows significantly lower amplitudes upon red-light stimulation than green-, blue-, or white-light stimulation.

### 3.3. Sensitizing Mouse RGCs to Express ChRmine for Non-Invasive Optogenetics

**Figure 3:**
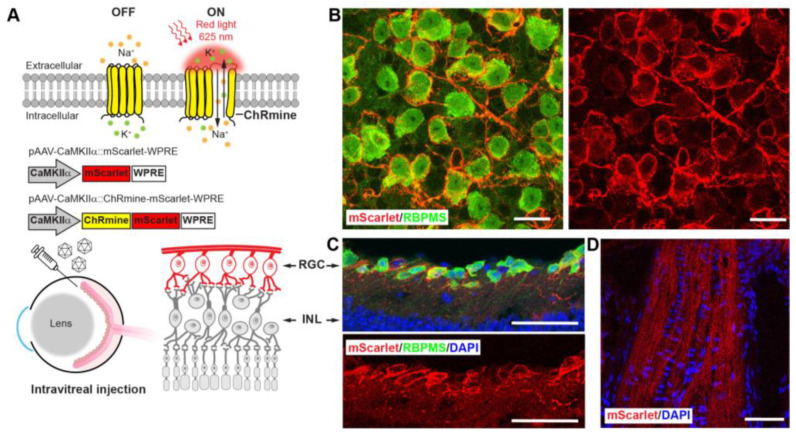
Transduction of mouse RGCs to express ChRmine using AAV delivery. (**A**) Schematic illustration of ChRmine optogenetic stimulation mechanism, AAV vector designs, and intravitreal injection of viral vector to transduce RGC layers. (**B**) Representative confocal images of whole-mount mouse retina. (**C**,**D**) Representative confocal images of the retina tissues (**C**) and optic nerve head (**D**) in mouse retinal cryosections at 21 days post-injection. ChRmine-mScarlet expression: red, RGC marker of RBPMS: green, nuclei stained with DAPI: blue. Scale bars: 20 μm (**B**) and 50 μm (**C**,**D**). WPRE, woodchuck hepatitis virus posttranscriptional regulatory element; RGC, retinal ganglion cells; INL, inner nuclear layer; RBPMS, RNA binding protein with multiple splicing factors; DAPI, 4′,6-diamidino-2-phenylindole.

Given the lower red-light sensitivity in mice, we anticipated less crosstalk from the endogenous red-light electrophysiological response. Therefore, we transduced mouse RGCs with the newly developed red-light opsin, ChRmine, through adeno-associated virus (AAV) intravitreal injection (Figure 3A). ChRmine is a pump-like cation-conducting channelrhodopsin. It exhibits large photocurrents with millisecond spike-timing fidelity and red-shifted light sensitivity. Intravitreal injection of AAV serotype 2 (AAV2/2) was chosen to efficiently transduce RGCs with minimal effect on other cell types [28,29].

AAV2/2 solutions carrying the gene of ChRmine fused to a red fluorescent reporter protein, mScarlet, under the control of a neuron-specific promotor, CaMKIIα: ChRmine-mScarlet (ChRmine), were intravitreally injected into mouse eyes. The same dose of AAV2/2 solutions carrying the same fluorescent reporter but without opsin, CaMKIIα: mScarlet (mScarlet), and PBS solutions were intravitreally injected in mouse eyes following the same procedures. The mice injected with the virus without opsin and with PBS served as the control groups. To confirm the gene expression, we inspected the retina and optic nerve at retinal cryosections and whole-mount retinal tissues using confocal microscopy (Figure 3B–D). To examine the cell-type targeting in the vision system of the AAV transduction strategy, we conducted the immunohistological staining of the retinal tissues with the antibody of the RNA binding protein with multiple splicing factors (RBPMS), a specific marker for RGCs. ChRmine expression was confirmed by observation of the fused mScarlet fluorescent protein in both retinal cryosections and wholemount retinal tissues (red channel in Figure 3B–D). By comparing the co-labeling of mScarlet and RBPMS (red and green channels, Figure 3B–D), we found a high percentage of overlayed mScarlet-positive and RBPMS-positive cells, indicating that RGCs were successfully transduced. We observed the membrane-targeted expression of the fused mScarlet in RGCs soma and optic nerves, as expected from the membrane–protein property of ChRmine.

### 3.4. ERG Responses of RGCs from Optogenetic Stimulation

**Figure 4:**
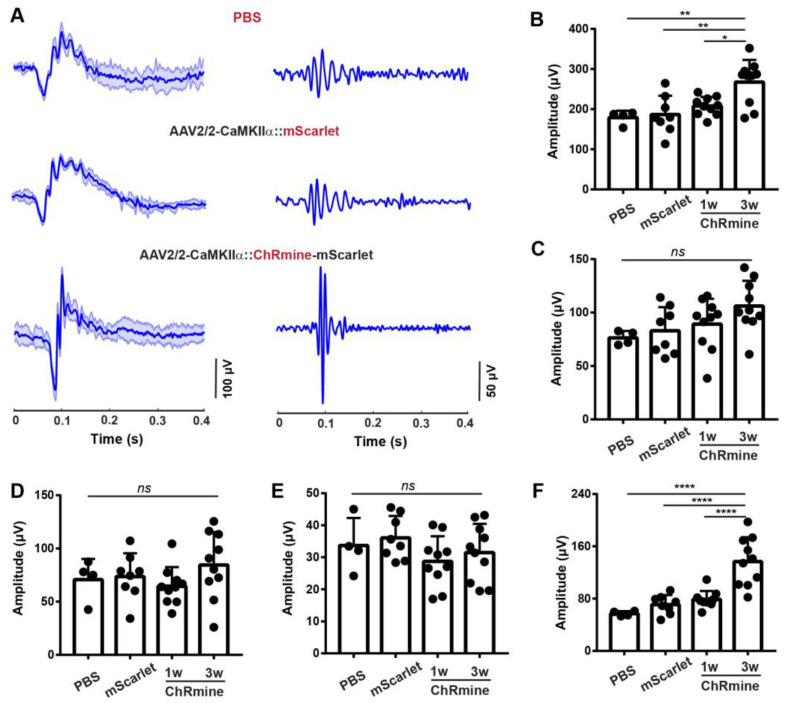
Scotopic flash ERG responses under optogenetic activation of RGCs. (**A**) Representative scotopic flash ERG waveforms (**left**) and OPs (**right**) under red-light stimulation. Three groups of mice are intravitreally injected with solutions of PBS, AAV2/2-CaMKIIα: mScarlet (mScarlet), and AAV2/2-CaMKIIα: ChRmine-mScarlet (ChRmine). The statistical quantification of the overall maximum response (**B**), a-wave (**C**), b-wave (**D**), NR (**E**), and OPs (**F**) of the scotopic flash ERG signals under red-light stimulations for different groups. The ERG signals in PBS and mScarlet mouse groups are collected at 2 weeks post-injection. The ERG signals in the ChRmine group are collected at 1 week (1 w) and 3 weeks (3 w) post-injection. PBS: N = 4 eyes, mScarlet: N = 8 eyes, ChRmine: N = 10 eyes. ns: not significant. * *p* < 0.05, ** *p* < 0.01, and **** *p* < 0.0001.

Specifically expressing ChRmine in RGCs offers unique access to dissect RGC’s electrophysiological response via ERG recordings. We conducted the scotopic flash ERG recording with a high-power fiber-coupled red (625 nm) LED in the ChRmine, mScarlet, and PBS mouse groups. Following the similar intravitreal injection strategy, mice RGCs were transduced to express CaMKIIα: ChRmine-mScarlet (ChRmine) or CaMKIIα:mScarlet (mScarlet), as well as only PBS injection (Figure 4A). The scotopic flash ERG responses across all three groups showed similar ERG patterns: an initial cornea-negative deflected a-wave, a sequential fast cornea-positive deflected b-wave, and a slow NR after the b-wave. OPs were observed on the rising part of the b-wave for all groups. Compared to the control groups (mScarlet and PBS), the OPs’ amplitudes of the ChRmine group were much higher.

Then we quantified the maximum amplitudes of the overall waveform, a-wave, b-wave, NR, and OPs of scotopic flash ERG responses under red-light stimulation (Figure 4B–F). For the mScarlet and PBS groups, we found no significant difference among all the examined ERG features, including the maximum response, a-wave, b-wave, NR, and OPs. This indicated that the fluorescent reporter, mScarlet expression does not affect the scotopic flash ERG responses under red-light stimulation.

All features of the scotopic flash ERGs low-frequency components, such as photoreceptor-mediated scotopic a-wave, b-wave, and NR, exhibited no significant difference among ChRmine, mScarlet, and PBS groups (Figure 4C–E). However, the high-frequency component, OPs, in the ChRmine group showed dramatically increased amplitude compared to the two control groups. The OPs in the ChRmine group showed an amplitude of 143 ± 38 µV, while the mScarlet and PBS groups presented amplitudes of 57 ± 4 µV and 73 ± 16 µV, respectively (Figure 4F). Consequently, the overall maximum ERG signals in the ChRmine group was much higher than those of the other groups with red-light stimulation (Figure 4B). We also found a gradual amplitude increase in OPs over time from 1-week post-injection to 3-week post-injection in the ChRmine group (Figure 4F). The timeline matches gene expression dynamics via AAV delivery. We suspect that this OPs amplitude increase corresponds to the ChRmine expression level. This observation further demonstrated RGCs’ contribution to OPs amplitude.

## 4. Conclusions

In this study, we report a platform technology to dissect neural electrophysiological dynamics with a custom-designed ERG recording system, integrated with a programmable non-invasive optogenetics stimulation device, and an automatic ERG data analysis approach. As a proof-of-concept application, we use this system to dissect the RGCs’ contribution to the scotopic flash ERG. Key features of ERGs signals, including a-wave, b-wave, NR, and OPs, can be classified efficiently in an unbiased fashion. With a remotely programmed light stimulation setup, we captured the scotopic flash ERG responses under red-, green-, blue-, and white-light stimulation. We also observed that the a-wave and OPs under red-light stimulation exhibited much lower amplitudes, while the b-wave and NR maintained similar amplitudes. To specifically isolate RGCs’ electrophysiological responses, we sensitized mouse RGCs to express the red-light-sensitive opsin, ChRmine, through AAV2/2 intravitreal injection. We found that ChRmine-expressing RGCs generated higher OPs, while the other ERG features presented no significant difference from the groups without opsin expression. We conclude that the ERG high-frequency component, OPs, is associated with red-light sensitivity and the RGC-mediated scotopic flash ERG responses. We envision that monitoring the OPs component in ERG signals can provide a new biosensing perspective for the early-stage diagnosis of RGC degenerative diseases, such as glaucoma.

## Data Availability

The data that support the findings of this study are presented within the manuscript and are available from the corresponding author upon request.
